# Interfering with emotional processing resources upon associative threat memory reactivation does not affect memory retention

**DOI:** 10.1038/s41598-019-40827-9

**Published:** 2019-03-12

**Authors:** Anastasia Chalkia, Lauranne Vanaken, Riet Fonteyne, Tom Beckers

**Affiliations:** 1 Centre for the Psychology of Learning and Experimental Psychopathology, Department of Psychology, KU Leuven, Leuven, Belgium; 2 Leuven Brain Institute, KU Leuven, Leuven, Belgium

## Abstract

Ample evidence suggests that memories enter a labile state upon retrieval, requiring reconsolidation processes in order to be retained. During this period of instability, various interventions can be applied to modify problematic memories. A novel behavioral intervention was designed, aimed at disrupting amygdala-based cognitive processing following the retrieval of a conditioned threat memory, in order to prevent its reconsolidation. We fear-conditioned participants on day 1, and reactivated their memory on day 2. Following reactivation, the reactivation plus emotional working memory task (R + EWMT) group completed an EWMT, while the reactivation only (RO) group served as a no-task control. On day 3, all participants were tested for memory retention, followed by a test for sensitivity to reinstatement. We observed successful acquisition and reactivation in fear-potentiated startle responding, skin conductance responding and US expectancies in both groups. Differential fear responding was fully preserved in the R + EWMT group relative to the RO group at the beginning of retention testing, and both groups were comparably sensitive to reinstatement. Thus, we failed to obtain any evidence that the execution of an EWMT after threat memory reactivation impairs reconsolidation. Further research is indicated to clarify whether threat memory reconsolidation can be disrupted by taxing relevant WM resources.

## Introduction

Experimental work in animals and humans has repeatedly shown that upon retrieval, memories can be brought back from a consolidated and inactive state into an active and labile state. While in this active state, memories are temporarily sensitive to suppression or modification, until they are restored in long term memory (for recent reviews see refs^1–3^). Various manipulations applied after memory retrieval can therefore block later memory expression, yielding specific amnesia for previously consolidated memories (e.g., refs^4,5^). The most widely established approach to interfere with such memories is through the administration of amnestic drugs upon memory retrieval, i.e., drugs that disrupt protein synthesis, a process that is proposed to be involved in the restabilization (or reconsolidation) of destabilized memories^6^ (but see ref.^7^). Reports of pharmacological disruption of reconsolidation in animals have involved the administration of anisomycin^6^, cycloheximide^8^, midazolam^9^, propranolol^10^, and others. While most of the aforementioned drugs are toxic at effective doses and not indicated for administration to humans, propranolol, a β-adrenergic receptor antagonist, is safe for human use and has provided for the first successful translation and observation of pharmacological reconsolidation blockade in humans^11^.

More recently, behavioral interventions aimed at interfering with reactivated memories have been established. One such example is memory updating, in which it is attempted to incorporate corrective information into the memory trace upon retrieval^12,13^. Schiller and colleagues^12^ first installed a threat memory in humans through differential fear conditioning, and then reactivated it 24 h later through a single unreinforced presentation of the CS+. Then, instead of administering an amnestic drug, the authors repeatedly presented subjects with the CS+ and the CS− in absence of reinforcement (i.e., extinction training). When subjects were tested for memory retention the next day, those that had received reactivation and extinction did not exhibit differential skin conductance responding (SCR), in contrast to the spontaneous recovery of differential responding observed in participants that had received extinction only (i.e., without prior memory reactivation). Moreover, when a portion of the subjects was tested again one year later, the latter but not the former exhibited sensitivity to reinstatement. The authors concluded that when new information is presented during the period of memory reconsolidation, it becomes incorporated into the existing memory trace and leads to its updating. However, the robustness of this reactivation-extinction effect is presently under debate, as replication attempts in animals and humans have yielded mixed results (for a review see ref.^14^; for a meta-analysis see ref.^15^).

An altogether different approach is to try to deploy behavioral techniques not to update reactivated memories, but to impair their reconsolidation, building on the same general rationale as that of pharmacological approaches to reconsolidation blockade. Just like on a neuronal level, reconsolidation of a reactivated memory may rely on protein synthesis, at a cognitive level, reconsolidation has been suggested to critically depend on active working memory processing (e.g., rehearsal)^16^. If cognitive processing is prevented during (part of) the reconsolidation process, through engagement in a working memory task that engages similar resources as those needed for the maintenance or processing of the reactivated memory, restorage and retention of the destabilized memory may thus be compromised. James and colleagues^16^ were the first to provide evidence that this rationale can indeed be harnessed to suppress visual trauma-like memories, by engaging visuo-spatial memory for a limited amount of time shortly after memory reactivation. In a week-long study, participants were exposed to a trauma film on the first day in order to create a strong traumatic experience that would evoke intrusions in the week to follow. The next day, a group of participants was presented with still film images as reminder cues in order to reactivate the memory, and following a 10-min break, was asked to play the computer game Tetris for 12 min. Control groups either received no reminder cues, no Tetris game play, or neither of the two. Seven days later, the group that received the memory reactivation followed by Tetris scored lower than the control groups on an intrusion-provocation task and reported having experienced a lower frequency of intrusions during the intervening week. In agreement with previous findings in the literature^11,17^, subjects’ declarative memory was fully preserved, as no differences between groups were found in the ability to visually or verbally recognize the traumatic film. The authors argued that processing the traumatic film, as well as playing Tetris, both of which are very visual in nature, requires visuo-spatial working memory (WM) resources. Therefore, playing Tetris on the second day while the traumatic memory was in an active state led to competition for WM resources and impaired the proper reconsolidation of visual aspects of the trauma film memory.

In addition to issues of replicability (see Discussion), it remains to be seen to what extent this rationale and approach can be generalized to non-visual emotional memories and memory indices other than intrusions, to connect it with the broader literature on emotional memory reconsolidation. Research on emotional memory reconsolidation in humans and animals does not typically employ intrusions and imagery, but often employs fear conditioning to install threat memories. Such associative threat memories are considerably less visual and imagery-rich in nature than trauma film memories. Investigating the generalizability of the *cognitive blockade* approach to preventing the reconsolidation of associative threat memories is the aim of the present paper.

In a three-day study, we established a threat memory on day 1 through differential fear conditioning, similar to previous memory updating and pharmacological studies of reconsolidation interference (e.g., refs^11,12^). On day 2, memory was reactivated through a single unreinforced presentation of the CS+, followed in the experimental group by the execution of a taxing emotional working memory task (EWMT) that has been previously shown to compete with and disrupt emotional processing^18^. Just like reconsolidation of visual emotional memories may critically depend on visuo-spatial WM resources, reconsolidation of non-visual associative threat memories may arguably require emotional working memory resources that are shared with other emotional stimulus processing tasks. Participants were tested for retention of the differential conditioned fear response on day 3, followed by a test of sensitivity to reinstatement. We hypothesized that execution of the EWMT after memory reactivation would disrupt differential fear-potentiated startle responding on day 3 in the experimental group, relative to a no-task control group. We did not expect any effects on differential SCR or US expectancies, given that pharmacological disruption of memory reconsolidation has also been shown to affect only the emotional aspect of memory, and not its declarative content^17,19,20^.

## Materials and Methods

### Pre-registration

The experimental procedures and statistical analysis plan were pre-registered on AsPredicted (https://aspredicted.org/mz7yh.pdf).

### Participants

Fifty-six healthy undergraduate students and community volunteers were recruited to participate in the study. Participants were first screened for the presence of any current or previous medical condition. Medical exclusion criteria included pregnancy, cardiovascular diseases, pulmonary diseases, neurological disorders, psychiatric disorders, other serious medical conditions, presence of an electronic implant (i.e., pacemaker), hearing problems, pain at the hand or wrist, blood phobia, and/or doctor’s request to avoid stressful situations. Participants with a score of 26 or more on the Anxiety Sensitivity Index (ASI) were also excluded (*n* = 4), as were individuals that did not complete the full three days of the experiment or experienced malfunctions with the psychophysiological equipment or recordings (*n* = 4). Finally, participants that exhibited zero or negative CS+/CS− differentiation in their fear-potentiated startle (FPS) responses over the last block of acquisition were also excluded (*n* = 8). The final sample thus included 40 participants (*n* = 20 per group) between 17 and 61 years of age (27 female, *M*_age_ = 22.88, *SD*_age_ = 7.71). The study was approved by the KU Leuven Social and Societal Ethics Committee, and all procedures were in accordance with the Declaration of Helsinki. All participants gave informed consent before the start of the study and were reimbursed with 30 euros or partial course credit for their participation.

### Stimuli

Two images of spiders taken from the International Affective Picture System (IAPS # 1200 and 1201)^21^ served as the conditioned stimuli (CSs). Allocation of the images to the role of CS+ and CS− was counterbalanced across participants. A mild electrical shock to the wrist served as the unconditioned stimulus (US). It was delivered through two disposable, pre-gelled 8-mm Ag/AgCl electrodes (Biopac Systems, Goleta, California) placed on top of the wrist of the dominant hand. All shocks were administered for a duration of 2 ms and shock delivery was controlled by a constant-current stimulator (DS7A, Digitimer, Hertfordshire, UK). Using a shock work-up procedure, participants were given the opportunity to select their own individual shock intensity level; once decided upon, this intensity was used throughout the experiment.

### Subjective Assessments

#### Ratings and US expectancies

Participants were asked to rate the CSs on valence and arousal using the Self-Assessment Manikins (SAM)^22^ prior to the start of the experiment, after the acquisition phase, and after completion of the whole experiment. Retrospectively, ratings of the distress induced by the US and the startle probes were obtained using an 11-point scale ranging from “not unpleasant” (0) to “very unpleasant” (10), as were ratings of the intensity and surprisingness of the US and the startle probes using a 5-point scale ranging from “light” (1) to “very strong” (5). These additional ratings were collected twice, once following the acquisition phase, and once after completion of the experiment. During the experiment, participants were asked on each trial to indicate their expectancy of the US using an 11-point scale ranging from “certainly no electric stimulus” (−5), over “uncertain” (0), to “certainly an electric stimulus” (5). This scale was presented at the bottom of the screen upon the onset of CS presentation. Participants had 7 s to indicate their expectancy.

#### Questionnaires

The Fear of Spiders Questionnaire (FSQ)^23^ was used to assess participants’ general level of spider phobia. The Anxiety Sensitivity Index (ASI)^24^ was used to assess the tendency to respond fearfully to anxiety-related symptoms. Finally, state and trait anxiety were measured using the State and Trait Anxiety Inventory (STAI-S/STAI-T)^25^.

### Psychophysiological Measures

#### Fear-potentiated startle (FPS)

FPS was measured through electromyography (EMG) of the right orbicularis oculi muscle. Two 4-mm Ag/AgCl electrodes filled with conductive electrolyte gel (Microlyte, Coulbourn Instruments, Holliston, Massachusetts) were placed 1 cm below the pupil and 1 cm below the lateral canthus, and a third (ground) electrode was placed on the forehead^26^. Acoustic startle probes (40 ms white noise, 100 dBA) were presented binaurally through headphones (Sennheiser HD 202). The EMG signal was sampled at 1000 Hz and amplified using an isolated bioamplifier with band-pass filter (Lablinc v75-04, Coulbourn Instruments) with a high pass filter of 13 Hz and a low pass filter of 500 Hz. The signal was rectified and smoothed online at a time constant of 20 ms, using a 4-channel integrator (Lablinc v76-24, Coulbourn Instruments). The analogue output was then digitized by a 16-bit AD converter (National Instruments, NI-6221, Austin, Texas). Offline processing was completed with PSPHA^27^. Blink amplitude was determined by subtracting a 20-ms baseline (0–20 ms following probe onset) from the peak response in a 21–200 ms window following probe onset. To standardize the data, means and standard deviations from the first testing session were used to calculate within-participant *Z*-scores.

#### Skin conductance response (SCR)

SCR was measured continuously at 100 Hz using a pair of disposable, pre-gelled 8-mm Ag/AgCl electrodes (Biopac Systems) attached to the palm of the non-dominant hand. The electrodes were connected to an isolated skin conductance coupler (LabLinc v71–23, Coulbourn Instruments). The skin conductance module was further connected to a 16-bit AD converter (National Instruments, NI-6221), which digitized the raw analogue SCR signal. Offline data extraction was completed with PSPHA^27^. SCR amplitude was determined by subtracting the average of a 2-s baseline (prior to stimulus onset) from the maximum response in a 0–7 s window following stimulus onset. This is an established approach for calculating SCR and has been extensively used in the past in our lab as well as others^17,28–35^. All responses were kept in the analysis, and SCR data were *Z*-transformed analogously to FPS responses.

### Behavioral Manipulation

**Figure 1:**
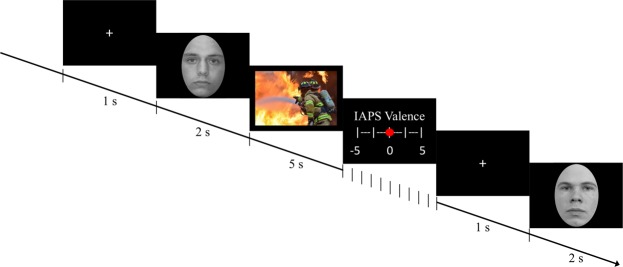
Illustration of the EWMT. Participants were asked to react to each face stimulus by pressing the right button if it was the same as the previous face and to press the left button if it was different from the previous one, and to rate the valence of the non-face pictures presented in between. Face stimuli were obtained from the Radboud Faces Database^37^. Non-face pictures were obtained from the International Affective Picture System^21^ (the picture depicted here is not an actual IAPS picture but a public-domain picture that is used for illustration purposes only).

The behavioral intervention that was used to interfere with emotional processing after memory reactivation was adapted from the emotional working memory task (EWMT) developed by King and Schaefer^18^. During this task, participants were presented with 128 trials comprised of the same sequence of stimuli (see Fig. 1). A white fixation cross was first presented on a black background for 1 s, followed by a neutral, black-and-white face stimulus for 2 s. An aversive or a positive IAPS image was then shown for 5 s, after which a rating scale was depicted on which participants were asked to rate the valence of the IAPS image. The scale ranged from very negative (−5), over neutral (0), to very positive (5). The rating scale did not have a fixed presentation time; each participant was allowed as much time as necessary to respond. Once the rating was registered, the next trial started. In addition to rating the IAPS pictures, participants were instructed to react to the face stimuli using a serial response (SR) box. Their task was to press the right button if the face presented on the current trial was the same as on the previous trial and to press the left button if the current face was different from that of the previous trial. Given the duration of the face stimuli presentations, participants had only 2 s to indicate their response. Note that face identity processing, like stimulus evaluation, has been shown to critically engage the amygdala^36^. Accordingly, face processing and emotional picture processing have been found to be mutually interfering in the EWMT^18^. Given that the amygdala is also critically involved in the formation and reconsolidation of associative threat memories, we hypothesized that the EWMT might be a suitable instrument for disrupting associative threat memory reconsolidation.

Eight neutral face stimuli with no extraneous, noticeable features were used during the task (four male and four female faces). They were enclosed in an oval frame measuring 7.5 × 5.5 cm and presented on a black background. Face stimuli were selected from the Radboud Faces Database^37^. 128 distinct IAPS images were selected on the basis of valence and arousal ratings^21^. The 64 aversive images depicted themes of mutilation, attack, weapons, accidents, and graphical medical conditions. On a 9-point scale, they had an average valence rating of 2.44 and an average arousal rating of 6.31. The 64 positive images depicted themes of food, money, nature, sports, and romance, and had a mean valence rating of 6.94 and a mean arousal rating of 5.97.

During 84 of the 128 trials, startle probes were presented either 2.5, 3.5, or 4.5 s after IAPS image onset. During the task, no more than 3 probed trials, no more than 3 IAPS images of the same valence, and no more than 4 trials including the same face were ever presented in a row. Participants were instructed to respond to each face stimulus as fast as possible while trying to not commit too many errors. Prior to the start of the task, a practice phase of 8 trials was given while the researcher stayed in the experimental cubicle to ensure that the instructions were fully comprehended by the participant.

### Procedure

The study was conducted on three consecutive days, each about 24 h apart (+/−3 h), with a 1-h duration of each session (protocol adapted from Kindt *et al*.^11^). Following attachment of the electrodes, each day began with a habituation phase where 10 Noise Alone (NA) startle probes were presented with a 15–25 s inter-trial interval (ITI) in order to stabilize startle responding. Order of trial presentations was completely randomized thereafter, with the restriction that no trial type was presented more than twice in a row.

#### Acquisition

During the first session, all participants underwent partly instructed differential acquisition. First, the ASI, STAI-S, STAI-T, FSQ, and SAM were administered. After electrode placement, the first session began with the shock work-up procedure. Upon its completion, the experimental instructions were presented. Participants were instructed that two images of spiders would be presented on the screen, and that one of them would sometimes be followed by a shock, while the other one would never be followed by a shock (following instructions of refs^11,17^). They were also told that they should learn to predict whether a shock would occur on the basis of the presented stimuli and they were instructed about how to indicate their US expectancy on the scale. Finally, it was stressed that they should remember the CS-US association for the next two sessions. At that point, the habituation phase (see above) commenced, followed immediately by the acquisition phase. Acquisition consisted of 8 CS+ (6 reinforced), 8 CS−, and 8 NA trials. CS trials had a duration of 8 s, with a variable 15–25 s ITI (*M* = 20 s). They were interspersed with the NA trials, which had a 40-ms duration (the duration of the startle probe). At the end of the session, participants were asked to provide their ratings, fill in the STAI-S again, and explicitly instructed to remember what they had learned during the session, as in the next 2 days they would be tested on it (cf. ref.^11^).

#### Reactivation and Behavioral Manipulation

At the start of the second session, the STAI-S was administered. After electrode placement, the instructions of the first day were reminded, in line with previous studies, in order to maximize prediction error (PE)^11,17^. An appropriate degree of PE during retrieval has been repeatedly demonstrated to be a necessary condition for memory reactivation (see Discussion). Upon completion of the habituation phase, 1 unreinforced CS+ trial was presented in order to reactivate the threat memory. Additionally, 1 NA trial was presented. Upon completion of the reactivation phase, the SCR and shock electrodes were removed whereas the EMG electrodes remained attached to measure startle responses during the EWMT. A 10-min break was inserted, during which the researcher opened an envelope to reveal the participant’s condition allocation, which was unknown to both parties up to this point. Those in the reactivation plus EWMT (R + EWMT) condition were then asked to complete the EWMT, while those in the reactivation only (RO) condition were left to sit quietly in the experimental cubicle doing nothing. The behavioral manipulation lasted for 25 min. At the end of the session, participants were again asked to fill in the STAI-S.

#### Retention and Reinstatement Test

At the start of the final session, all participants filled in the STAI-S. After electrode placement, the instructions only stated that the same images would be presented on the screen as on the previous days (cf. ref.^11^). Following the habituation phase, a retention test was presented which contained 12 CS+, 12 CS−, and 12 NA trials, all unreinforced and randomized as before. In addition to being a retention test, this phase also served to extinguish conditioned responding. 19 s after the last extinction trial, 3 unsignalled USs were administered, followed after another 18 s by the presentation of another 4 CS+, 4 CS−, and 4 NA trials, again all unreinforced and in random order, to test for sensitivity to reinstatement. At the end of the session, participants were asked to once again fill in the STAI-S and provide CS and US ratings.

### Statistical Analyses

After transformation, FPS and SCR responses were averaged over blocks of two trials and then analyzed using repeated-measures analysis of variance (rm-ANOVA) with Group (R + EWMT, RO) as a between-subjects factor and Block (First, Last) and Cue (CS+, CS−, [NA]) as within-subjects factors. US expectancies were also analyzed using a rm-ANOVA with Group as a between-subjects factor and Trial (First, Last) and Cue as within-subjects factors. FSQ, ASI, STAI-T, STAI-S, SAM and retrospective distress ratings were analyzed using independent-samples *t*-tests. STAI-S and SAM ratings were also analyzed using a rm-ANOVA with Group as a between-subjects factor and Moment as a within-subjects factor. Finally, accuracy, reaction times and startle responses during the EWMT were analyzed using paired-samples *t*-tests. Outliers were determined for each day (Z-score > 3) and replaced by linear trend at point. Greenhouse–Geisser corrections were applied in case of violation of sphericity. An alpha level of 0.05 was set for all analyses, which were executed using JASP version 0.8.6^38^.

## Results

### Demographics and Subjective Assessments

The R + EWMT group did not differ from the RO group in age (R + EWMT: *M* = 23.2, *SD* = 9.15, RO: *M* = 24.55, *SD* = 6.12; *t*(38) = 0.55, *p* = 0.59) or spider fear (FSQ) (R + EWMT: *M* = 35.1, *SD* = 37.59, RO: *M* = 29.5, *SD* = 30.64; *t*(38) = −0.52, *p* = 0.61). The groups also did not differ in baseline anxiety levels as indexed by state (STAI-S) (R + EWMT: *M* = 36.5, *SD* = 11.69, RO: *M* = 33.85, *SD* = 8.93; *t*(38) = −0.81, *p* = 0.43) or trait anxiety (STAI-T) (R + EWMT: *M* = 36.4, *SD* = 10.94, RO: *M* = 36.25, *SD* = 8.16; *t*(38) = −0.05, *p* = 0.96). Finally, the individually selected US intensity was comparable across groups (R + EWMT: *M* = 31.8, *SD* = 19.75, RO: *M* = 25.8, *SD* = 14.06; *t*(38) = −1.11, *p* = 0.28), as was the subjective rating of the selected US intensity (R + EWMT: *M* = 8.11, *SD* = 1.05, RO: *M* = 8.1, *SD* = 0.79; *t*(38) = −0.02, *p* = 0.99). The groups did differ in gender distribution and education levels. Specifically, in the RO group women (*n* = 18) greatly outnumbered men (*n* = 2), whereas in the R + EWMT group gender was more evenly distributed (women: *n* = 9, men: *n* = 11) (*X*^2^ (1, *N* = 40) = 9.23, *p* = 0.002). In terms of education level, the RO group contained more highly educated participants than the R + EWMT group (*X*^2^ (2, *N* = 40) = 6.68, *p* = 0.04). Finally, a small but significant difference was also detected in anxiety sensitivity (ASI) measured prior to the start of the experiment, with participants in the R + EWMT group reporting slightly higher anxiety sensitivity than those in the RO group (R + EWMT: *M* = 13.85, *SD* = 5.99, RO: *M* = 10.35, *SD* = 4.51; *t*(38) = −2.09, *p* = 0.04).

Ratings of the CSs on valence and arousal did not differ between the groups at any of the three time points. Additionally, ratings of the intensity of the US and the startle probes or the surprise or distress they provoked did not differ between the groups either, except for the rated intensity of the shock following acquisition (R + EWMT: *M* = 2.90, *SD* = 0.45, RO: *M* = 3.20, *SD* = 0.41; *t*(38) = 2.21, *p* = 0.03) and the rated intensity of the startle probes following completion of the whole experiment (R + EWMT: *M* = 1.73, *SD* = 0.56, RO: *M* = 2.30, *SD* = 0.57; *t*(38) = 3.102, *p* = 0.004). Both of those were rated higher in the RO group, perhaps due to women experiencing the shock and startle stimuli as somewhat more intense than men.

### Acquisition and Memory Retrieval

**Figure 2:**
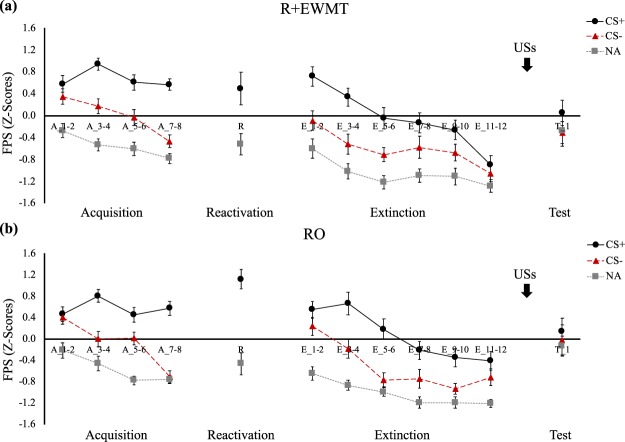
Mean FPS (*Z*-score) across all phases of the experiment for (**a**) the R + EWMT group and (**b**) the RO group. Error bars represent standard error of the mean.

**Figure 3:**
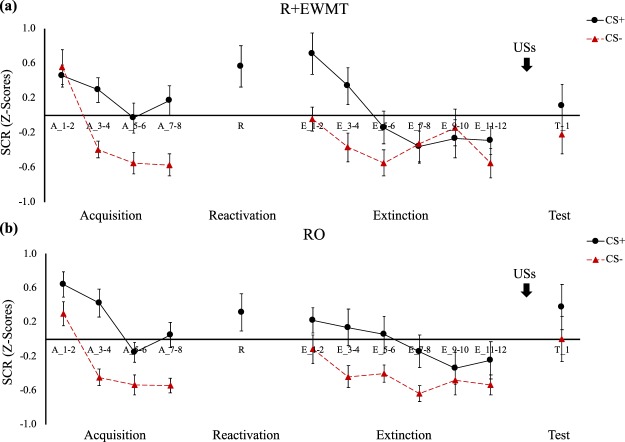
Mean SCR (*Z*-score) across all phases of the experiment for (**a**) the R + EWMT group and (**b**) the RO group. Error bars represent standard error of the mean.

**Figure 4:**
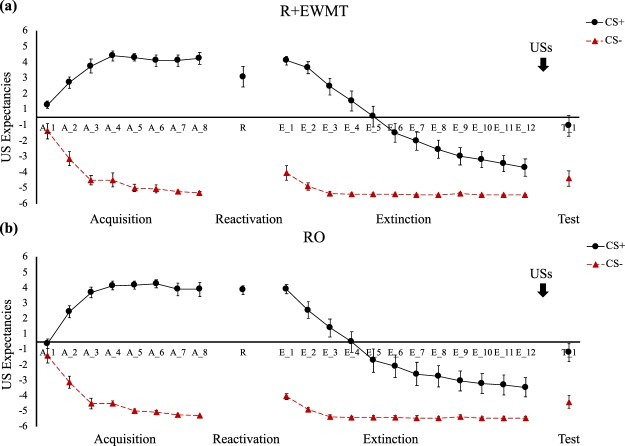
Mean US expectancies across all phases of the experiment for (**a**) the R + EWMT group and (**b**) the RO group. Error bars represent standard error of the mean.

Differential threat learning on day 1 was achieved across all three outcome measures. Participants developed higher FPS responses to the CS+ than to the CS− by the end of acquisition (cue * block, *F*(1,38) = 24.69, *p* < 0.001, *η*_*p*_^2^ = 0.39), and this pattern did not differ between the groups (group * cue * block, *F*(1,38) = 1.04, *p* = 0.31, *η*_*p*_^2^ = 0.03). (Fig. 2). A similar pattern emerged when the CS+ was compared to NA (cue * block, *F*(1,38) = 14.49, *p* < 0.001, *η*_*p*_^2^ = 0.28; group * cue * block, *F*(1,38) < 1, *n.s*.). Similarly, in SCR, CS+/CS− differentiation increased from the first to the last block of acquisition (cue * block, *F*(1,38) = 6.70, *p* = 0.01, *η*_*p*_^2^ = 0.15), in both groups comparably (group* cue * block, *F*(1,38) = 2, *p* = 0.17, *η*_*p*_^2^ = 0.05) (Fig. 3). Lastly, we also observed successful conditioning of differential US expectancies (cue * trial, *F*(1,31) = 187.27, *p* < 0.001, *η*_*p*_^2^ = 0.86), which did not differ between groups (group * cue * trial, *F*(1,31) < 1, *n.s*.) (Fig. 4).

On the second session, the threat memory was successfully retrieved, as indicated by higher FPS responding on the CS+ than the NA trial (main effect of cue, *F*(1,38) = 36.51, *p* < 0.001, *η*_*p*_^2^ = 0.49), with no differences between the groups (group * cue, *F*(1,38) = 1.73, *p* = 0.20, *η*_*p*_^2^ = 0.04) (Fig. 2). In SCR, comparing the retrieval trial for both groups required conducting a Mann-Whitney *U* test instead of an independent samples *t*-test as responding in the RO group deviated from normality. The two groups did not significantly differ from each other in SCR on the retrieval trial (*U*(38) = 170, *p* = 0.43) (Fig. 3). Also in US expectancy, there were no differences in responding between the groups during memory retrieval (*t*(36) = 1.09, *p* = 0.29) (Fig. 4).

### Emotional Working Memory Task

#### Valence, reaction times and accuracy

Ratings for the aversive images (*M* = −3.19, *SD* = 0.85) were significantly lower than ratings for the positive images (*M* = 1.96, *SD* = 0.78; *t*(19) = 16.92, *p* < 0.001), as expected. Mean reaction times (RT; in ms) and mean accuracy (percentage of correct responses) for the face classification responses were analyzed by preceding IAPS picture (positive, negative) and face identity (same or different face as on previous trial). Participants took longer to decide whether a face was the same or different from the previous one following a negative IAPS picture (*M* = 1054.56, *SD* = 132.63) than following a positive picture (*M* = 1021.64, *SD* = 153.68; *t*(19) = −2.29, *p* = 0.03). Decision times did not significantly differ between same face trials (*M* = 1034.87, *SD* = 143.94) and different face trials (*M* = 1043.07, *SD* = 143.93; *t*(19) = −0.59, *p* = 0.56). The pattern was exactly opposite for error rates. Face classification accuracy following a negative IAPS picture (*M* = 87.95, *SD* = 8.79) did not differ from the accuracy following a positive IAPS picture (*M* = 89.55, *SD* = 9.11; *t*(19) = 1.09, *p* = 0.29), but participants were more accurate on different face trials (*M* = 92.07, *SD* = 8.40) than same face trials (*M* = 85.52, *SD* = 11.64; *t*(19) = −2.54, *p* = 0.02).

#### Startle potentiation

To directly compare our startle data to those of King and Schaefer^18^, we processed the data in the same way as they did, implying that trials with an excessive baseline (higher than 3 *SD* from the mean) and trials with low blink amplitude (smaller than 20% of the mean) were discarded, data were subsequently *Z*-transformed, and *Z*-scores were then converted to *T*-scores [(*Z*-score * 10) + 50]. Note that this processing, applied here for consistency with King and Schaefer^18^, deviates from the standard procedure of processing startle data that was applied to the fear-potentiated startle data from the actual fear conditioning procedure (see above).

Across trials, startle amplitudes were higher in the presence of negative IAPS pictures (*M* = 50.54, *SD* = 1.40) than in the presence of positive IAPS pictures (*M* = 48.93, *SD* = 1.56; *t*(19) = 2.54, *p* = 0.02). In total, 88 startle probes were presented during the EWMT (4 during the practice, and 84 during the actual task). Given that startle responding is sensitive to habituation, we split the startle data into two blocks (first, last). In the first block, startle amplitudes were again significantly higher for the negative stimuli (*M* = 53.22, *SD* = 2.51) than for the positive stimuli (*M* = 50.61, *SD* = 1.53; *t*(19) = 3.40, *p* = 0.003). In the second block, startle amplitudes did not differ anymore (*t*(19) = 0.89, *p* = 0.39).

### Retention and Reinstatement Testing

#### Fear-Potentiated Startle

To investigate whether our manipulation interfered with reconsolidation, we first compared the last block of acquisition to the first block of retention testing, where a significant group by cue by block interaction emerged (*F*(1,38) = 4.34, *p* = 0.04, *η*_*p*_^2^ = 0.10), implying that differential FPS responding changed over time, but this pattern of responding differed between the groups (Fig. 2). Follow-up paired-sample *t*-tests on each group separately revealed that in the RO group CS+ responding at the beginning of the retention test was not reliably different from the end of acquisition (end of acquisition: *M* = 0.58, *SD* = 0.59, beginning of retention: *M* = 0.55, *SD* = 0.69; *t*(19) = 0.13, *p* = 0.90). Startle amplitudes to the CS− however increased from the end of acquisition to the start of retention testing (end of acquisition: *M* = −0.70, *SD* = 0.50, beginning of retention: *M* = 0.24, *SD* = 0.76; *t*(19) = 5.10, *p* < 0.001), indicating enhanced fear responding to the safe stimulus on the retention test. This increase was evident only in the RO group, as in the R + EMWT group, responding to the CS+ and to the CS− did not change significantly (*t*(19) = 0.74, *p* = 0.48; *t*(19) = 1.74, *p* = 0.10, respectively).

On the retention test (first block of extinction), differential FPS responding was significant (main effect of cue, *F*(1,38) = 23.21, *p* < 0.001, *η*_*p*_^2^ = 0.38), and different between the groups, (group * cue, *F*(1,38) = 4.53, *p* = 0.04, *η*_*p*_^2^ = 0.11). In contrast to our hypothesis, the RO group showed significantly less discrimination between CS+/CS− (CS+: *M* = 0.55, *SD* = 0.69, CS−: *M* = 0.24, *SD* = 0.76; *t*(19) = 1.61, *p* = 0.12) than the R + EMWT group (CS+: *M* = 0.72, *SD* = 0.78, CS−: *M* = −0.09, *SD* = 0.80; *t*(19) = 6.30, *p* < 0.001). Of note, this was due to an increase in CS− responding in the former group. Differential responding diminished from the beginning to the end of retention testing, suggesting successful extinction (cue * block, *F*(1,38) = 5.76, *p* = 0.02, *η*_*p*_^2^ = 0.13). Again, the decline in differential responding differed significantly between groups (group * cue * block, *F*(1,38) = 5.69, *p* = 0.02, *η*_*p*_^2^ = 0.13), due to the intact differential CS+/CS− responding in the R + EWMT group at the beginning of extinction, which was less pronounced in the RO group. There was no significant group difference in the degree of differential responding by the end of extinction, (group * cue, *F*(1,38) < 1, *n.s*.).

Finally, comparing the last block of extinction to the first trial of reinstatement testing, a main effect of cue was observed (*F*(1,38) = 5.37, *p* = 0.03, *η*_*p*_^2^ = 0.12), which was not significantly modulated by trial (cue * trial, *F*(1,38) < 1, *n.s*.) or group (group * cue * trial, *F*(1,38) < 1, *n.s*.). In contrast to our hypothesis, then, our intervention did not significantly impair threat memory retention or prevent its reinstatement after extinction as measured by FPS responding.

#### Skin Conductance

In SCR, when the last block of acquisition was compared to the first block of retention testing, there was an increase in responding over time (main effect of block, *F*(1,38) = 13.35, *p* < 0.001, *η*_*p*_^2^ = 0.26), while differential responding remained intact (main effect of cue, *F*(1,38) = 33.66, *p* < 0.001, *η*_*p*_^2^ = 0.47), with no significant interaction effects (cue * block, *F*(1,38) < 1, *n.s*.) (Fig. 3). Similar to previous phases of the experiment, once again, groups did not significantly differ (group * cue * block, *F*(1,38) < 1, *n.s*.).

On the first block of the retention test, as hypothesized, differential SCR responding remained intact, with participants exhibiting greater responses for the CS+ than the CS− (main effect of cue, *F*(1,38) = 16.11, *p* < 0.001, *η*_*p*_^2^ = 0.30). No difference between the groups could be identified (group * cue, *F*(1,38) = 2.33, *p* = 0.14, *η*_*p*_^2^ = 0.06). Furthermore, from the beginning to the end of retention testing, no changes in differential responding were detected (cue * block, *F*(1,38) = 2.46, *p* = 0.13, *η*_*p*_^2^ = 0.06). No reliable group differences were observed in the lack of extinction (group* cue * block, *F*(1,38) = 1.63, *p* = 0.21, *η*_*p*_^2^ = 0.04).

Finally, when comparing the last block of extinction training with the first trial of reinstatement testing, differential responding was still observed (main effect of cue, *F*(1,38) = 7.74, *p* = 0.008, *η*_*p*_^2^ = 0.17). This pattern of responses was not affected by the reinstatement procedure (cue * block, *F*(1,38) < 1, *n.s*.), nor did it differ between the groups (group * cue * block, *F*(1,38) < 1, *n.s*.). Analogous to our FPS findings, and as we hypothesized, our intervention did not affect the retention of threat in SCR, nor its sensitivity to reinstatement.

#### US Expectancies

Differential US expectancies changed from the end of acquisition to the beginning of retention testing (cue * trial, *F*(1,36) = 13.38, *p* < 0.001, *η*_*p*_^2^ = 0.27) (Fig. 4). Follow-up *t*-tests showed that participants still expected the CS+ to be followed by the US (end of acquisition: *M* = 3.58, *SD* = 1.88, beginning of retention: *M* = 3.51, *SD* = 1.23; *t*(38) = 0.34, *p* = 0.74). However, at the beginning of the retention test, their expectancy to receive a US after the CS− also increased (end of acquisition: *M* = −4.85, *SD* = 0.37, beginning of retention: *M* = −3.88, *SD* = 1.64; *t*(38) = −3.73, *p* < 0.001), a pattern similar to the one we observed for FPS. However, in the US expectancies, this pattern did not differ between groups (group * cue * trial, *F*(1,36) = 2.06, *p* = 0.16, *η*_*p*_^2^ = 0.05).

On the first trial of retention testing, as expected, differential US expectancies were preserved (main effect of cue, *F*(1,37) = 550.95, *p* < 0.001, *η*_*p*_^2^ = 0.94), with no significant group differences (group * cue, *F*(1,37) < 1, *n.s*.). A standard extinction pattern was observed from the beginning to the end of retention testing (cue * trial, *F*(1,37) = 129.17, *p* < 0.001, *η*_*p*_^2^ = 0.78), which did not differ between groups (group * cue * trial, *F*(1,37) < 1, *n.s*.).

Comparing the last trial of extinction to the first trial of reinstatement testing, a significant cue by trial interaction emerged (*F*(1,38) = 11.56, *p* = 0.002, *η*_*p*_^2^ = 0.23), and this pattern did not differ between groups (group * cue * trial, *F*(1,37) < 1, *n.s*.). Follow-up *t*-tests showed that US expectancies for both the CS+ and the CS− increased from the end of extinction to the beginning of reinstatement testing (CS− end of extinction: *M* = −4.88, *SD* = 0.40, reinstatement: *M* = −3.95, *SD* = 1.97; *t*(39) = −3.20, *p* = 0.003), with the greatest increase in CS+ responding (CS+ end of extinction: *M* = −3.08, *SD* = 2.60, reinstatement: *M* = −0.63, *SD* = 2.80; *t*(39) = −6.12, *p* < 0.001). As for FPS and SCR, we observed a retention of differential US expectancies and a differential reinstatement after extinction.

## Discussion

The present study investigated the effect of a non-invasive, behavioral manipulation aimed to disrupt the reconsolidation of a reactivated conditioned threat memory. Participants were trained in a differential fear conditioning paradigm with two fear-relevant CSs on the first day. Twenty-four hours later, a brief retrieval session was conducted where one CS+ trial was presented, unreinforced, to reactivate the threat memory. Shortly after, the R + EWMT group completed a demanding working memory task intended to interfere with emotional processing, while the RO group sat quietly in the experimental cubicle. On the third day, memory retention was examined, followed by a test of reinstatement. We hypothesized that performing the EWMT during the time-limited process of reconsolidation would lead to competition for cognitive processing resources, and might therefore induce amnesia for the conditioned threat memory in the R + EWMT group on day 3. We did not find any evidence in support of this hypothesis, as both groups showed mostly similar retention of conditioned responding and a similar return of fear (sensitivity to reinstatement) after extinction. The only group difference in retention that we observed (in FPS) was opposite to our predictions.

A variety of reasons may explain our failure to find the hypothesized result. First, our amnestic intervention might have not been sufficiently strong or specific. Execution of the EWMT was supposed to compete for shared emotional processing resources with memory reconsolidation, but this might not have happened – it is unclear what the benchmark should be for sufficient task load to create successful competition. Second, our reactivation procedure may have failed to destabilize the conditioned threat memory and bring it back into an active state in which it would become sensitive to amnestic interventions. The boundary conditions for emotional memory destabilization that are emerging from recent research are narrow and extensive; for example, we might not have achieved the optimal degree of prediction error during retrieval for inducing memory reactivation^28^. Third, gender differences or stress might have influenced our results. Finally, our results might indicate that the logic of disrupting cognitive processing during reconsolidation as a means to impair memory retention and return of fear does not apply beyond the very concrete and visual trauma-like memories targeted in previous research, or may simply not be a robust phenomenon at all. We will discuss those possibilities in turn.

James and colleagues^16^ proposed that engagement of working memory resources after memory reactivation interferes with reconsolidation, hence we endeavored to identify an interference task that would actively tax relevant working memory resources. King and Schaefer^18^ demonstrated that the EWMT as used here puts a considerable load on working memory and disrupts (supposedly amygdala-based) emotional processing. Note however that our accuracy and FPS data during execution of the EWMT critically deviate from those reported by King and Schaefer^18^. Whereas they found that participants were slower and less accurate in face categorization after a negative IAPS picture, we observed an effect of preceding IAPS picture on the speed of face categorization only. In terms of accuracy, we witnessed similarly solid performance following positive and negative pictures. This may reflect a ceiling effect in our sample, as the accuracy following negative pictures (88%) was considerably higher than what was reported in the original study^18^ (82.5%). One might take this to indicate that participants’ attention was attracted to a somewhat lesser extent by the IAPS pictures in the current study. However, whereas in King and Schaefer^18^, startle responding to negative IAPS pictures was attenuated, which they argued to indicate that the necessity to keep face stimuli online depleted the amygdala from the necessary resources to process the IAPS pictures’ emotional content, we observed significantly higher startle responding to negative than to positive IAPS pictures, which in combination with the reduced face classification speed following negative pictures clearly argues against a lack of processing of the IAPS pictures’ emotional content. As such, it seems warranted to conclude that the EWMT implied a load on emotional processing resources, be it that the balance between IAPS picture processing and face processing may have been somewhat different in the present study than in King and Schaefer^18^. This renders an explanation of the present results in terms of insufficient strength or specificity of our amnestic intervention unlikely.

With respect to a potential lack of memory destabilization, ample research in the past 20 years has revealed important boundary conditions on the ability of memory retrieval to induce destabilization. A consolidated memory trace has to be brought back into an active state in order for it to be susceptible to interference^39^. However, if a memory trace became active every time that a memory was retrieved then memory would arguably be too malleable to be adaptive. It has been proposed that memory destabilization and reconsolidation serve the function of allowing for the updating of memories, so as to make them agreeable with the current environment and/or situation^40^. Therefore, only when there appears to be a need for memory updating at the time of memory retrieval, would the memory trace be triggered into destabilization and a subsequent need for reconsolidation^41,42^. More formally, a *prediction error (PE)*, or a discrepancy between what is expected on the basis of the retrieved memory and what actually occurs, is hypothesized to be a critical requirement for the induction of threat memory reconsolidation upon memory retrieval^17,43^. Furthermore, it has been shown that the degree of PE at the time of retrieval that will induce reconsolidation is highly constrained^28^. An insufficient degree of PE will cause mere memory retrieval, without concomitant memory destabilization, whereas an excessive degree of PE will lead to the formation of a new memory trace rather than the induction of reconsolidation^44,45^. One could therefore argue that in the present study the reactivation procedure may have simply failed to induce destabilization of the conditioned threat memory trace. Note however that the fear conditioning protocol and the memory reactivation procedure that we used (employing a single unreinforced CS+) were identical to those used in previous research that successfully demonstrated sensitivity to amnestic pharmacological interventions upon reactivation^11,17,33,34^ (but see refs^46,47^). Although this makes an explanation of the current results in terms of a lack of memory destabilization unlikely, in the absence of an independent neural marker of memory destabilization in humans we cannot definitively rule out a lack of memory destabilization.

We observed a significant difference in gender distribution between the two groups, which could in principle also have influenced our results. Sex differences have been found in brain activation during fear conditioning and extinction^48^ and hormonal fluctuations are suggested to be responsible for these variations^49^. Further, and specifically in extinction, sex differences are influenced by current phase of the menstrual cycle in female subjects, with early-cycle women and men expressing stronger extinction memory relative to mid-cycle women^50^. In our study, we indeed detected differences in extinction between the two groups, with the RO group showing significantly less discrimination between CS+/CS− on the first block of extinction due to enhanced responding to the CS−. As women prevailed in the RO group, this finding is in line with previous research showing that women, and in particular those using hormonal contraceptives, discriminate less between fearful and safe stimuli^51^. In our study, we did not take record of hormonal contraceptive use, phase of the menstrual cycle or hormone levels/secretions. To evaluate possible effects of the unequal gender division across conditions, we therefore conducted additional post-hoc analyses that either included female participants only or used gender as a covariate. The results of those analyses did not differ in any meaningful way from the ones reported here.

Stress has variably been shown to have either an enhancing or an impairing effect on memory, depending on factors like the duration of stress or the timing of stress induction (prior to memory formation, during consolidation, or prior to retrieval of the memory trace). While there is ample research regarding effects of stress on other memory systems, data on the effects of stress on threat memory reconsolidation in humans are sparse, and findings are mixed. Meir Drexler and colleagues^52^ administered cortisol, a stress hormone, prior to memory reactivation in healthy men, and found an enhancement of memory reconsolidation, whereas in another study, they found the opposite effect, namely, impairment of reconsolidation following a behavioral stress induction conducted before reactivation^53^. Remarkably, cortisol administration had no effect on the reconsolidation of threat memories in women^54^. It seems possible that differences in stress may have had some influence on our results. STAI-S scores were higher in the R + EWMT group than the RO group after our manipulation on day 2, although they did not increase from before to after the manipulation in the former group; the effect was driven by a decrease in state anxiety in the RO group (see Supplementary Information). Given that the R + EWMT group was perhaps more stressed than the control group, this may have induced a relative enhancement in the reconsolidation of their threat memory, countering any impairment induced by the reduction in cognitive resources during reconsolidation. We can only speculate about this possibility here, as our study did not include a direct measure of stress. Future research might include objective indices of the stress response (e.g., cortisol assays) in conjunction with subjective ratings to better illuminate a possible influence of stress on threat memory reconsolidation.

The original report by James *et al*.^16^ held great promise for a simple, easy-to-use intervention to disrupt memory following its reactivation. A few follow-up investigations that build on the same rationale have also reported positive results^55–57^, but on closer inspection, those follow-up studies mostly suffer from critical limitations, such as failing to disentangle effects of game play on reconsolidation or initial consolidation (for a discussion see ref.^58^). Also, those replications all involved the same lab, leaving a need for independent replication of the effect. The present study was the first to use a working memory task at the time of memory retrieval to try to block the reconsolidation and attenuate the expression of a conditioned threat memory. We were unable to find evidence for the hypothesis that performing an emotional working memory task during a period of memory lability, by competing for emotional processing resources, can disrupt threat memory reconsolidation. Although there may be extraneous reasons for that failure, there is of course also the distinct possibility that the alleged mechanism of cognitive interference upon memory reactivation simply does not generalize beyond visual, trauma-like memories, or that this approach may not be very robust to begin with. Further work will be needed to elucidate whether threat memory reconsolidation can indeed be interfered with by targeting relevant working memory processes. Such work is vital for the future development of much-needed, evidence-based protocols exploiting reconsolidation interference to treat anxiety disorders.

## Supplementary information

Supplementary Information

## Data Availability

Partial, de-identified data are available on the Open Science Framework [https://osf.io/4vsr3/]. Additional data requests may be addressed to the corresponding author.
