# Supplementary material for: Interfering with emotional processing resources upon associative threat memory reactivation does not affect memory retention
Source: Sci Rep. 2019 Mar 12;9:4175. doi: 10.1038/s41598-019-40827-9 (PMC6414694; doi:10.1038/s41598-019-40827-9)
Supplement: Supplementary file 1 — Supplementary Information [file 41598_2019_40827_MOESM1_ESM.pdf]

Supplementary Information

Interfering with emotional processing resources upon associative threat memory reactivation does not affect memory retention

Anastasia Chalkia, Lauranne Vanaken, Riet Fonteyne, Tom Beckers*

***Correspondence:** tom.beckers@kuleuven.be

Additional analyses – State anxiety

In state anxiety, a general decrease over time was observed from prior to versus after completion of the experiment (main effect of time, $F(3.24,123.25) = 3.32$, $p = .02$, $\eta_p^2 = 0.08$), and this effect did not differ between the groups (group * time, $F(3.24,123.25) < 1$, *n.s.*). Yet, state anxiety was hypothesized to differ between groups from before versus after the manipulation on the second day of the experiment, as the R+EWMT group completed a working memory task with aversive stimuli that ought to raise anxiety levels. We therefore compared STAI-S scores from before to after the manipulation and observed that state anxiety tended to differ between the two groups (group * moment, $F(1,38) = 3.41$, $p = .07$, $\eta_p^2 = 0.08$). Considering the differences in gender distribution between the two conditions, gender was taken as a covariate, and doing so, the interaction between moment and condition became significant ($F(1,38) = 5.39$, $p = .03$, $\eta_p^2 = 0.13$). Following up, a closer look revealed that, as expected, state anxiety significantly decreased in the RO group (before: $M = 31.95$, $SD = 7.76$, after: $M = 28.75$, $SD = 6.54$; $t(19) = 3.23$, $p = .004$), while surprisingly, it did not change significantly in the R+EWMT group (before: $M = 34.40$, $SD = 8.04$, after: $M = 35.30$, $SD = 9.85$; $t(19) = -0.45$, $p = .66$). Finally, we

examined STAI-S scores independently before and after the manipulation on day 2. Group averages were comparable before the manipulation ($t(38) = -0.98$, $p = .33$), and significantly different afterwards, with the R+EWMT group scoring higher in state anxiety than the control group ($t(38) = -2.48$, $p = .02$).
